# Corticosterone Excess-Mediated Mitochondrial Damage Induces Hippocampal Neuronal Autophagy in Mice Following Cold Exposure

**DOI:** 10.3390/ani9090682

**Published:** 2019-09-14

**Authors:** Bin Xu, Limin Lang, Shize Li, Jianbin Yuan, Jianfa Wang, Huanmin Yang, Shuai Lian

**Affiliations:** College of Animal Science and Veterinary Medicine, Heilongjiang Bayi Agricultural University, Daqing 163319, China; xubin@byau.edu.cn (B.X.); langlanglimin@163.com (L.L.); byndlsz@163.com (S.L.); byndeyhy@163.com (J.Y.); wjflw@sina.com (J.W.)

**Keywords:** cold stress, CORT excess, hippocampal neuronal, mitochondria damage, autophagy

## Abstract

**Simple Summary:**

In this study, the phenomenon of ‘autophagy’ was demonstrated in the hippocampus following cold exposure. Persistent neuronal stimulation of the hippocampus after corticosterone (CORT) treatment induced mitochondrial damage and autophagy by activating the AMPK/mTOR signaling pathway, which did not rely on glucocorticoid receptors (GRs). The phenomenon in the hippocampus of the cold stress mice was also a sex-related difference in the response to cold stress; the phenomenon of autophagy was more severe in males. These findings provided a new understanding of the underlying mechanisms of the cold stress response, which may influence the selection of animal models in future stress-related studies.

**Abstract:**

Cold stress can induce autophagy mediated by excess corticosterone (CORT) in the hippocampus, but the internal mechanism induced by cold stress is not clear. In vivo, male and female C57BL/6 mice were stimulated in 4 °C, 3 h per day for 1 week to build the model of cold sress. In vitro, hippocampal neuronal cell line (HT22) cells were incubated with or without mifepristone (RU486) for 1 h, then treated with 400 μM cortisol (CORT) for 3 h. In vivo, autophagy was measured by western blotting. In vitro, monodansylcadaverine staining, western blotting, flow cytometry, transmission electron microscopy, and immunofluorescence were used to characterize the mechanism of autophagy induced by excess CORT. Autophagy was shown in mouse hippocampus tissues following cold exposure, including mitochondrial damage, autophagy, and 5’ AMP-activated protein kinase (AMPK)/mammalian target of rapamycin (mTOR) pathway activation after CORT treatment. Autophagy did not rely on the glucocorticoid receptor. In addition, autophagy in male mice was more severe. The study would provide new insight into the mechanisms and the negative effect of the cold stress response, which can inform the development of new strategies to combat the effects of hypothermia.

## 1. Introduction

Stress is the normal physiological response of the body to environmental or psychological changes, and numerous studies have reported that chronic stress induces physiological and pathophysiological damage [1]. Cold exposure is a common problem as a stress to humans who live or work in extreme environments, and this exposure can induce cold stress and a large amount of studies have indicated cold stress have a negative influence on organism [2,3]. Many studies about stress have reported that stress activates the hypothalamic-pituitary-adrenal (HPA) axis response, and releases stress hormones such as corticosterones (CORT) to resist the negative effects of stress. However, chronic stimulation from stress can induce over-activation of the HPA axis and cause CORT secretory abnormalities [4]. The hippocampus is the first target tissue of CORT, and chronic stress causing excessive CORT may result in irreversible damage to the hippocampus [5,6]. A previous study has demonstrated that chronic stress could induce oxidative stress, neuronal apoptosis, and other nonspecific reactions in the brain of mice, which influence the CA1 and CA3 region functions of the hippocampus manifested as emotion, memory, adaptability, and learning-ability dysfunctions [5]. In our previous study, neuroinflammation [7,8], increased oxidation, and neuronal loss following acute and chronic cold exposure in the CA1 and CA3 regions of mice hippocampus were reported; as we know, CA1 and CA3 are the key regions of hippocampus, however, the underlying mechanism is not yet known, and whether the above phenomenon is associated with an excess of CORT has not been established.

Autophagy is the primary endogenous process of eradicating damaged cell organelles or unused proteins. Autophagy can be induced by internal and external factors including nutrient and energy deficiencies, oxidation stress, and mitochondrial damage [2,3,9]. Some studies have reported that autophagy is active following stimulation by stress during tissue repair [10]. In our previous study, the phenomenon of oxidation stress induced by excess CORT was shown to occur in the hippocampus of mice after cold exposure. However, whether there is an association between autophagy and cold exposure has not yet been determined.

Chronic cold stress has previously been reported to break the homeostasis of the hippocampus, which may be a major factor in neurodegenerative diseases, but the mechanism of the homeostasis imbalance in the hippocampus following cold exposure is not understood. In the present study, we characterized the phenomenon of autophagy in the hippocampus of mice following cold exposure and identified the mechanism, as well as consequences, of autophagy and CORT exposure in hippocampal HT-22 cells after CORT exposure.

## 2. Materials and Methods

### 2.1. Animals and the Experimental Design

Adolescent male and female C57BL/6 mice (5-weeks-old; 22–24 g) were purchased from Charles River (Beijing, China). The conditions of cold exposure have been previously described [11]. Each group was pre-fed in a climatic chamber at an ambient temperature of 24 ± 2 °C and 40% relative humidity, under a 12/12 h light/dark cycle (lights on from 8:00 am to 8:00 pm), with free access to food and water for 1 week. The animals were divided into four groups: the cold exposure male (CEM) group; the cold exposure female (CEF) group; the room temperature male (RTM) group; the room temperature female (RTF) group. The CEM and CEF groups were transferred to a climatic chamber at 4 °C for 3 h per day, and then back to room temperature between the hours of 8:00 am and 8:00 pm. The process of chronic cold exposure continued for 7 days. All experimental procedures were approved by the Management Committee of the Experimental Animal Center of Heilongjiang Bayi Agricultural University.

### 2.2. Hippocampus Tissue Protein Extraction

The method to extract the protein was referenced to our previous study [7]. In short, total hippocampal protein was extracted with 150 μL radio immunoprecipitation assay (RIPA) buffer (Beyotime, Hangzhou, China) containing 15 mM *phenylmethylsulfonyl fluoride* (PMSF; Beyotime, Hangzhou, China). The protein concentration was determined using the Enhanced BCA Protein Assay Kit (Beyotime, Hangzhou, China) according to the manufacturer’s instructions. The samples were stored at −80 °C for western blot analysis.

### 2.3. Cell Culture and Reagents

Mouse hippocampal HT22 cells were a generous gift from Professor Liu (College of Veterinary Medicine, Jilin University, Jilin, China) and maintained in the recommended culture conditions. The cells were maintained in Dulbecco’s Modified Essential Medium supplemented with 10% fetal bovine serum (Gibco, Carlsbad, CA, USA) at 37 °C in a 5% CO_2_ humidified incubator. The cells were grown in a monolayer and routinely passaged two or three times a week.

### 2.4. CORT and RU486 Treatment

CORT and RU486 (Sigma-Aldrich, St. Louis, MO, USA) were dissolved in dimethyl sulfoxide (DMSO) (Solarbio, Beijing, China). HT-22 cells were incubated with or without RU486 for 1 h, then the cells was treated with 400 μM CORT to build the model of CORT exposure referenced from previous studies [12], for 3 h to obtain an excess CORT model.

### 2.5. Annexin V-FITC/propidium Iodide (PI) Staining

After CORT incubated, HT22 cells were digested with trypsin and collected. To quantitate cell death, the cells were stained with fluorescein isothiocyanate (FITC)-labeled propidiumiodide (PI) and annexin V (Beyotime) for 30 min. Fluorescence labeling was analyzed by two-color flow cytometry. Annexin V and PI emissions were detected in the FL1 and FL2 channels of a flow cytometer (CytoFLEX FCM; Beckman, Brea, CA, USA) using emission filters of 488 and 532 nm, respectively reference from our previous study [13].

### 2.6. Adenosine Triphosphate (ATP)/Adenosine diphosphate (ADP)/Adenosine Monophosphate (AMP) Analyses

After CORT treatment, the levels of ATP/ADP/AMP of HT22 cells were determined by high-performance liquid chromatography using a Sepax Bio-C_18_ column (4.6 mm i.d. × 250 mm; 5 μm and 200 angstrom beads) and a UV detector at a wavelength of 254 nm (bandwidth:16 nm) as previously described [14].

### 2.7. Measurement of the Mitochondrial Membrane Potential

The mitochondrial membrane potential (∆Ψm) of the cells after CORT treated was measured. In a nutshell, HT22 cells were maintained in 20 mm confocal petri dishes (NEST, Jiangsu, China) until cell attachment and use CORT treatment, the ∆Ψm was detected using a mitochondrial membrane potential assay kit, according to the manufacturer’s instructions, and then the cells were viewed using a laser scanning confocal microscope (TCS SP2, Leica, Wetzlar, Germany).

### 2.8. Monodansylcadaverine (MDC) Staining

HT22 cells were maintained in 20 mm confocal petri dishes (NEST) and after CORT treatment, autophagy in HT22 cells was assessed after staining with an MDC assay kit following the manufacturer’s instructions (Solarbio, Beijing, China). All cells were viewed using a laser scanning confocal microscope.

### 2.9. Cell Protein Extraction

Total cell proteins were extracted from cell samples treated with 400 μM CORT using 100 μL RIPA buffer containing 10 mM PMSF. The samples were stored at −80 °C for western blot analysis. Protein concentration was determined using the Enhanced BCA Protein Assay Kit according to the manufacturer’s instructions.

### 2.10. Cell Nuclear Protein Extraction

Nuclear proteins were extracted from the cell sample treated with 400 μM CORT using the Nuclear and Cytoplasmic Protein Extraction Kit (Beyotime, Hangzhou, China), and the protein concentration was determined using the Enhanced BCA Protein Assay Kit according to the manufacturer’s instructions.

### 2.11. Western Blot Analysis

Approximately 30 μg of hippocampus and cell total protein was separated by sodium dodecyl sulfate-polyacrylamide gel electrophoresis and transferred to a polyvinylidene fluoride membrane (0.22 μm and 0.45 μm; Millipore, Darmstadt, Germany). The membranes were blocked in 5% nonfat milk in TBST (Tris-HCl, NaCl, and Tween 20) for 1 h at room temperature, then incubated overnight at 4°C with the following primary antibodies: glucocorticoid receptor (GR) (#24050-1-AP, 1:6000), cytochrome C (#10993-1-AP, 1:1000), B-cell lymphoma 2 (Bcl-2) (#12789-1-AP, 1:2000), Bcl-2-associated X (Bax) (#50599-2-Ig, 1:6000), Nucleoporin p62 (p62) (#18420-1-AP, 1:1000), LC3 (#14600-1-AP, 1:1000), beclin1 (#11306-1-AP, 1:1000), β-actin (#14395-1-AP, 1:15,000) or lamin B1 (#12987-1-AP, 1:1000) (all from Proteintech) and phospho-glucocorticoid receptor (P-GR) (Ser211), extracellular signal–regulated kinases (ERK) (#4161, 1:1000), phospho-ERK (Thr202/Tyr204) (#4377, 1:1000), AMPK (#9158, 1:1000), phospho-AMPK (Thr172) (#5759, 1:1000) were used as internal controls. The membranes were rinsed with TBST five times for 10 min each and incubated with the following secondary antibodies: horseradish peroxidase (HRP)-conjugated Affinipure goat anti-mouse Immunoglobulin G (IgG) (H+L) (SA00001, 1:8000; Proteintech) or HRP-conjugated Affinipure goat anti-rabbit IgG (H+L) (SA00001-1, 1:8000; Proteintech) for 1.5 h at room temperature. Membranes were then rinsed as previously mentioned, and treated with Chemiluminescent HRP Substrate (Millipore, Burlington, MA, USA), which was detected using a chemiluminescence detector (Bio-Rad, Hercules, CA, USA). The expression of each protein was measured using Image Lab software (Bio-Rad).

### 2.12. Cell Immunofluorescence

HT22 cells were seeded on slides and incubated for 24 h at 37 °C, followed by incubation with 200 μM CORT for 24 h; they were then fixed with 4% paraformaldehyde and seeded on poly-L-lysine-coated coverslips. The cells were permeabilized with 0.3% Triton X-100, blocked with 3% bovine serum albumin, and incubated overnight at 4 °C with antibodies against GR (Proteintech; 24050-1-AP, 3:100), followed by CL488-conjugated Affinipure donkey anti-mouse IgG (H+L) (Proteintech; SA00006-5, 1:200). Nuclei were stained with 4′,6-diamidino-2-phenylindole and the slides were viewed with a laser scanning confocal microscope.

### 2.13. Transmission Electron Microscopy

After CORT treatment, the cells were washed twice with ice cold PBS, and fixed with 2.5% glutaraldehyde in 0.15 mM sodium cacodylate at 4 °C overnight, then postfixed in 2% osmium tetroxide. All samples were dehydrated in ethanol and embedded in epoxy resin. Then, ultrathin sections (80 nm) of adherent cells were obtained using an ultramicrotome (EM UC7, Leica, Wetzlar, Germany). The sections were counterstained with uranyl acetate and lead citrate and observed using a JEM SX 100 electron microscope (Jeol, Tokyo, Japan) to capture images reference from our previous study [13].

### 2.14. Statistical Analysis

Statistical analyses were performed using Prism v.7.0 software (GraphPad Inc., La Jolla, CA, USA). Values are expressed as mean ± SD. Statistical comparisons were performed across different treatment groups (room temperature and cold exposure) and between sexes (male and female) by two-way analysis of variance and in vitro data were analyzed by one-way analysis of variance. *p* < 0.05 was considered statistically significant.

## 3. Results

### 3.1. The Expression of Activated GR in the Hippocampus of Mice Following Cold Exposure

**Figure 1:**
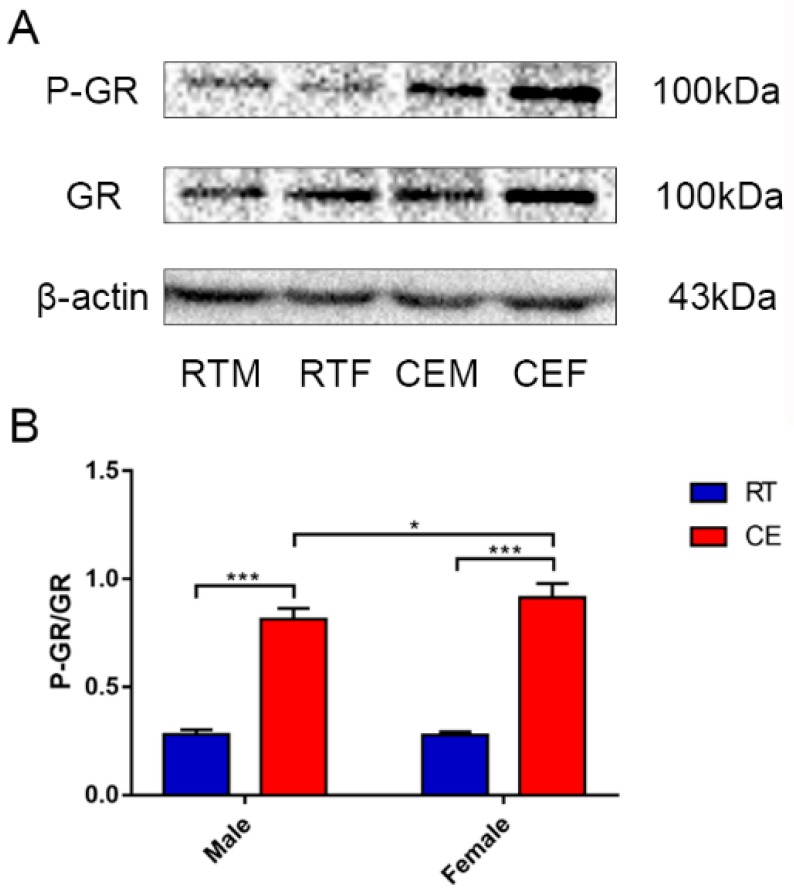
The relevant expression of the glucocorticoid receptor (GR) and phospho(P)-GR (**A**), and β-actin expression in hippocampus lysates as assessed by western blotting in each group. Expression levels were quantitated by measuring band intensities using Image Lab software. The graphs indicate the results of densitometric analyses using the expression ratios of (**B**) P-GR/GR. Data were between room temperature male vs. cold exposure male (CEM), room temperature female vs. cold exposure female (CEF), and CEM vs. CEF, and were analyzed by two-way analysis of variance. The results are expressed as the mean ± SD (n = 6) of four independent experiments analyzed by two-way analysis of variance. * *p* < 0.05; *** *p* < 0.001.

The expression of GR and the activated phosphorylated (P)-GR form were measured in hippocampus tissue lysate of each group using western blotting (Figure 1A). The blots showed an increase in the expression level of GR Ser211-phosphorylation both in the CEM and CEF groups (Figure 1B). Additionally, the results increase observed in the CEM group was significantly greater than the CEF group.

### 3.2. Autophagy in the Mouse Hippocampus Following Cold Exposure

**Figure 2:**
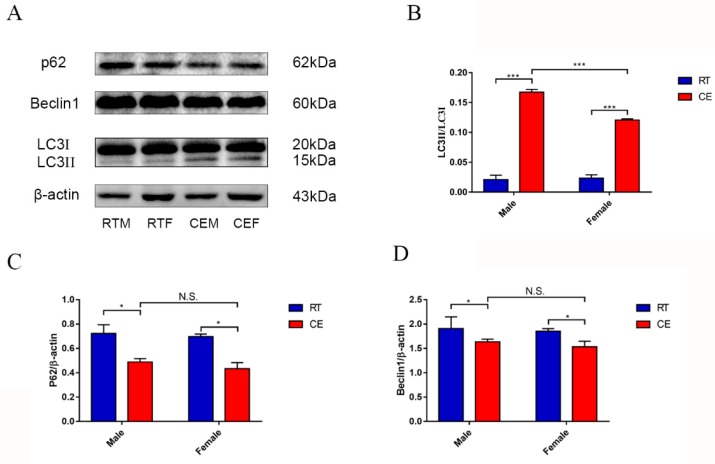
The relevant marker expressions of autophagy in hippocampus lysates as assessed by western blotting and relative density analyses (**A**). The expressions of p62, ceclin1, LC3, and β-actin in each group. The expression levels were quantitated by measuring band intensities using Image Lab software. The graphs indicate densitometric analyses using the expression ratios of (**B**–**D**) p62/β-actin, beclin1/β-actin, and LC3 II/ LC3 I. Data were compared between room temperature male vs. cold exposure male (CEM), room temperature female vs. cold exposure female (CEF), and CEM vs. CEF, and were analyzed by two-way analysis of variance. The results are expressed as the mean ± SD (n = 6) of four independent experiments analyzed by two-way analysis of variance. NS: not significant; * *p* < 0.05; *** *p* < 0.001.

The relevant protein expressions of autophagy p62, beclin1, and LC3 were measured in the hippocampal tissue of each group by western blotting (Figure 2A). The results showed an increase in the protein expression level of LC3II in the CEM and CEF groups (Figure 2B). Additionally, the increase in the CEM group was significantly greater than that in the CEF group. The expressions of p62 and beclin1 were decreased in the CEM and CEF groups (Figure 2C,D).

### 3.3. The Activation of GR after CORT Treatment with or without RU486 Incubation of HT22 Cells

**Figure 3:**
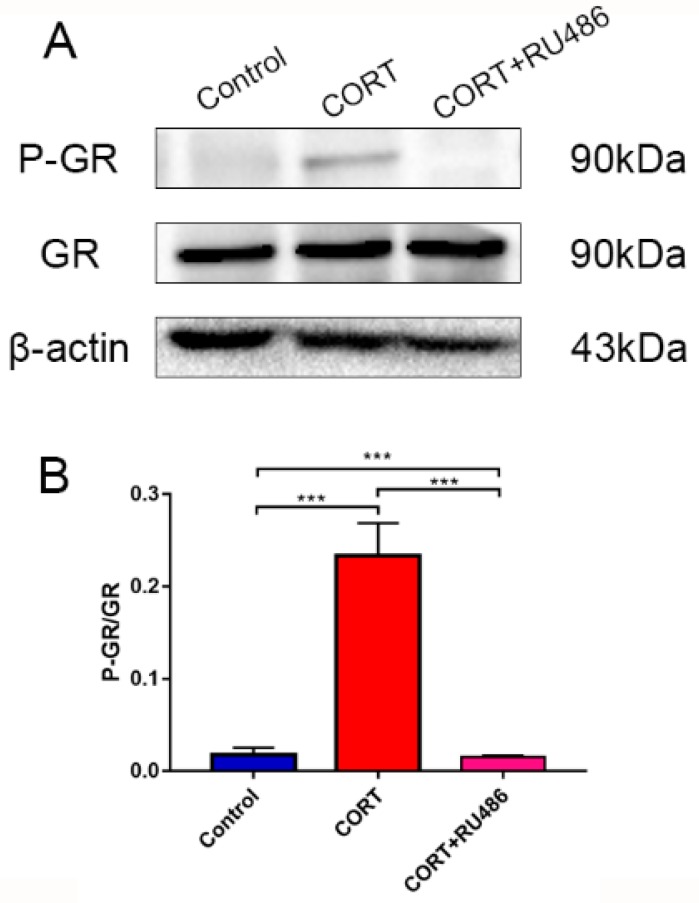
The relevant expression of protein in HT22 cell lysates after cortisol treatment for 3 h with RU486 or not as assessed by western blotting and relative density analysis (**A**) GR, P-GR, and β-actin expression in each group. Expression levels were quantitated by measuring band intensities using Image Lab software. The graphs indicate densitometric analyses using the expression ratios of (**B**) P-GR/GR. The results are expressed as the mean ± SD (n = 3) in each independent experiment analyzed by one-way analysis of variance. *** *p* < 0.001.

**Figure 4:**
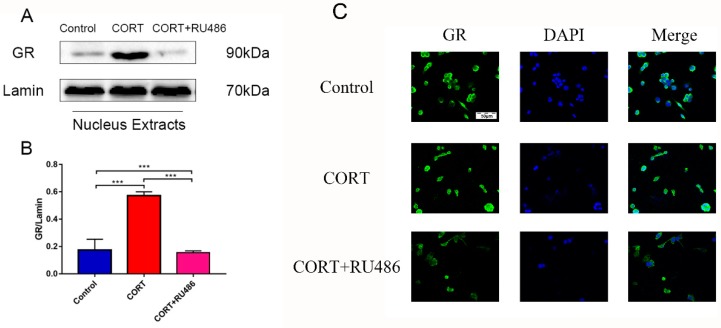
The relevant expression of nuclear proteins in HT22 cell nuclear lysates after cortisol (CORT) treatment for 3 h with or without RU486 as assessed by western blotting and relative density analyses. (**A**) The glucocorticoid receptor (GR) and lamin expressions in each group. Expression levels were quantitated by measuring band intensities using Image Lab software. The graphs indicate densitometric analyses using the expression ratios of (**B**) GR/lamin. The results are expressed as the mean ± SD (n = 3) in each independent experiment analyzed by one-way analysis of variance. *** *p* < 0.001. The GR localization was detected by immunofluorescence staining with or without 400 μM CORT treatment for 3 h with or without RU486 treatment.

To build the CORT excess model, 400 μM of CORT was used, as previously described and our previous study [13]. The activated level of GR was measured by western blotting (Figure 3A; Figure 4A) and the levels of the GR were measured by immunofluorescence (Figure 4C). The level of GR Ser211-phosphorylation expression of the total cell lysate by western bolting was measured after CORT treatment for 3 h with or without preincubation with RU486. The results indicated that the level of P-GR was upregulated in the CORT treatment group, but was not significantly upregulated with RU486 pretreatment (Figure 3B). The expression of GR in nuclear extracts indicated that this protein was significantly up-regulation on HT22 cells after treated with 400 μM CORT. However, there was no change in the RU486 + CORT group after identical treatment (Figure 4B). The immunofluorescence results showed that it was remarkable increase in GR which nuclear localization after CORT treatment for 3 h in HT22 cells, but with no change in the RU486 + CORT group (Figure 4C). 

### 3.4. Mitochondrial Damage and Autophagy Induced by Excess CORT in HT22 Cells

**Figure 5:**
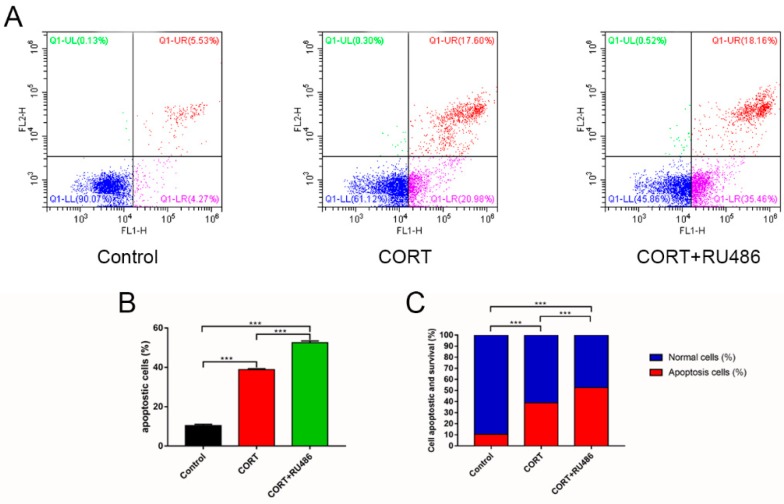
HT22 cells were treated with 400 μM cortisol (CORT) for 3 h after with or without RU486 treatment. HT22 cell apoptosis was detected by flow cytometry after labeling with fluorescein isothiocyanate (FITC)-annexin V (FITC-A, x-axis) and propidium iodide (y-axis) (**A**). The ratio of cell apoptosis after CORT treatment (**B**), and the proportions of normal and dead cells after CORT treatment (**C**). The results are expressed as the mean ± SD (n = 3) in each independent experiment analyzed by one-way analysis of variance. *** *p* < 0.001.

**Figure 6:**
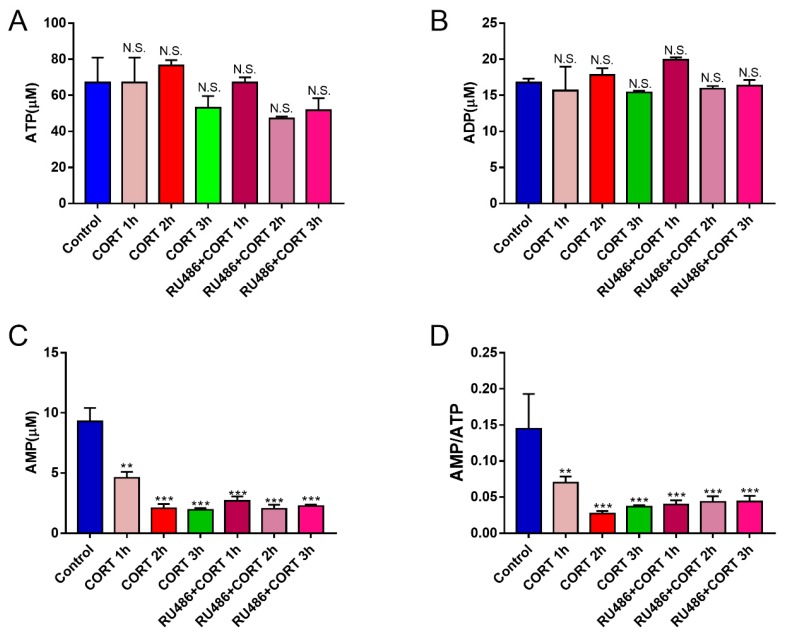
The ATP, ADP, and AMP levels (**A**–**C**) and the trend of ATP, ADP, and AMP levels in HT22 cells after cortisol treatment at different times (0–3 h) were measured, and the ratio of AMP to ATP was determined (**D**). The results are expressed as the mean ± SD (n = 3) in each independent experiment analyzed by one-way analysis of variance. NS: not significant; ** *p* < 0.01; *** *p* < 0.001.

**Figure 7:**
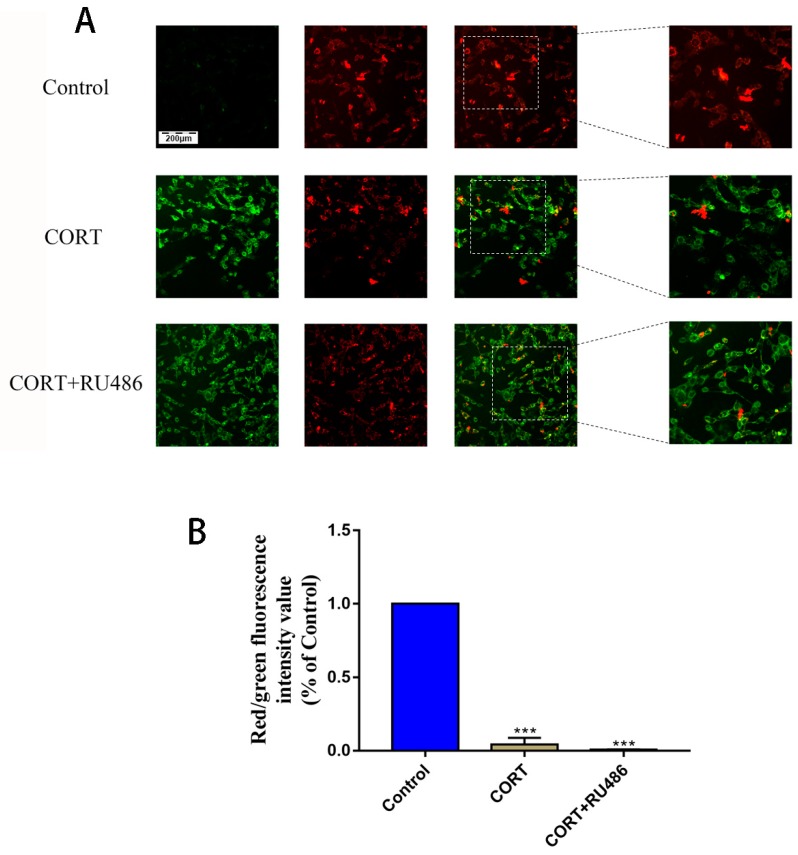
∆Ψm of HT22 cells was measured by JC-1 staining with 400 μM cortisol treatment for 3 h with or without RU486. The red color denotes JC-1 aggregates, and the green color denotes monomers (**A**). The bar graph shows the ratio of red to green fluorescence at every time point (**B**). Data were compared with control and analyzed by one-way analysis of variance. The results are expressed as the mean ± SD (*n* = 3) in each independent experiment; *** *p* < 0.001.

**Figure 8:**
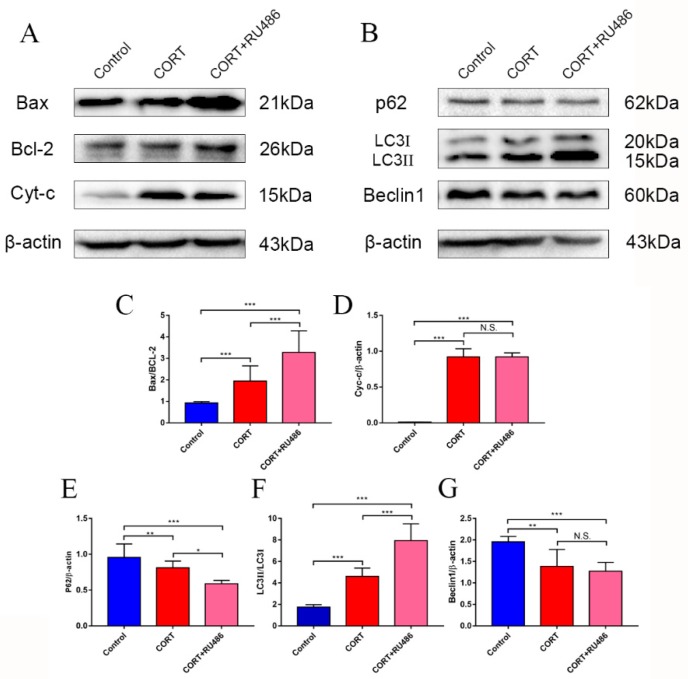
The relevant expression of autophagy markers in HT22 cell lysates after cortisol treatment for 3 h as assessed by western blotting and relative density analyses. (**A**,**B**) Bax, Bcl-2, Cyt-c, p62, LC3, beclin1, and β-actin expression in each group. Expression levels were quantitated by measuring band intensities using Image Lab software. The graphs indicate densitometric analyses using the expression ratios of (**C**) Bax/Bcl-2, (**D**) Cyt-c/β-actin, (**E**) p62/β-actin. (**F**) LC3II/ LC3I, and (**G**) beclin1/β-actin. The results are expressed as the mean ± SD (n = 3) in each independent experiment analyzed by one-way analysis of variance. NS: not significant; * *p* < 0.05; ** *p* < 0.01; *** *p* < 0.001.

**Figure 9:**
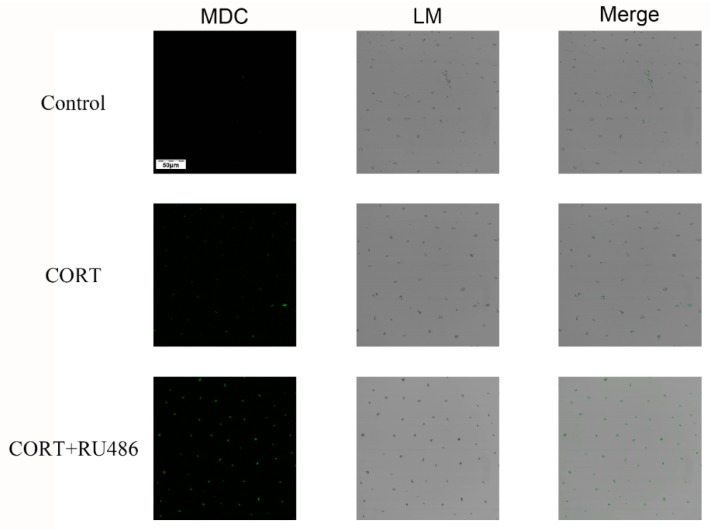
The phenomenon of autophagy in HT22 cells was observed by monodansylcadaverine (MDC) staining after 400 μM CORT treatment for 3 h with or without RU486 treatment. The green color denotes MDC staining.

**Figure 10:**
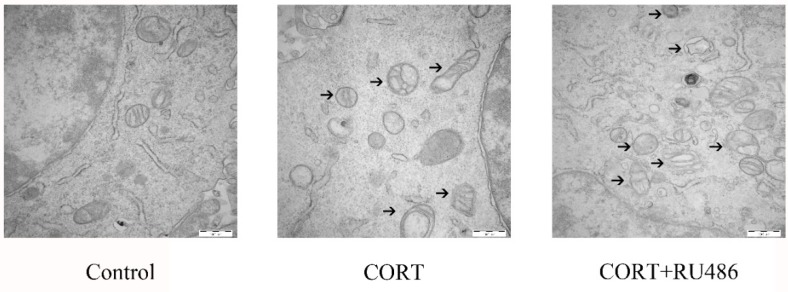
Mitochondrial damage due to autophagy in HT22 cells was observed by electron microscopy after 400 μM cortisol treatment for 3 h with or without RU486 treatment. The damaged mitochondria were indicated with black arrows.

The mitochondrial damage induced by excess CORT was measured by FITC-labeled PI and annexin V staining (Figure 5A), using ATP/ADP/AMP ratios/levels (Figure 6A–D), mitochondrion integrity (Figure 7), western blotting (Figure 8), MDC staining (Figure 9), and transmission electron microscopy (Figure 10). FITC-labeled PI and annexin V staining showed that the number of apoptosis events in HT22 cells after CORT treatment increased when compared with the control (Figure 5B) and further increased in the CORT + RU486 group. The number of cells after treatment with CORT is shown in Figure 5C. The results of measuring the level of ATP/ADP/AMP demonstrated that the levels of ATP and ADP were unchanged in each group (Figure 6A,B) when compared with the control, and the AMP level was decreased at 1 h in the CORT-treated and CORT + RU486 treated groups (Figure 6C). Moreover, the trend of AMP/ATP ratios showed a significant change (Figure 6D) after treatment with 400 μM CORT, which was remarkable in the CORT + RU486-treated groups. The results of the ∆Ψm assay indicated that the ∆Ψm of HT22 cells was significantly reduced after CORT (400 μM) treatment, and this reduction was greater when the cells were treated with RU486 before CORT treatment (Figure 7). The expressions of the apoptosis-related proteins, Bax, Bcl-2, and Cyt-c, were measured by western blotting (Figure 8A). The Bax:Bcl-2 ratio and Cyt-c were all significantly increased in the CORT treatment group, and it was remarkably increased in the CORT + RU486 group (Figure 8C,D). The relevant proteins of autophagy LC3II were significantly increased, and p62 and beclin1 were decreased after CORT treatment. MDC staining demonstrated that the number of positive cells increased after CORT treatment, and there was a remarkable increase in the CORT + RU486 group (Figure 9). Transmission electron microscopy directly confirmed that mitochondrial damage and autophagy occurred in the CORT treatment group, and that this damage was more severe in the CORT + RU486-treated group.

### 3.5. The Potential Mechanism of Autophagy Induced by Excess CORT in HT22 Cells

**Figure 11:**
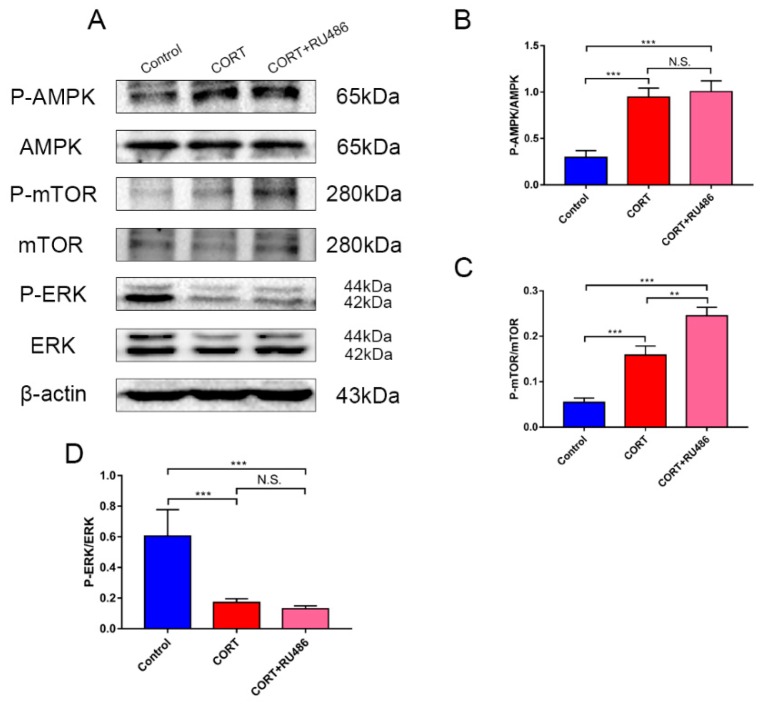
The relevant signaling pathway expression of key proteins in HT22 cell lysates after cortisol treatment for 3 h as assessed by western blotting and relative density analyses. (**A**) AMPK, P-AMPK, mTOR, P-mTOR, ERK, P-ERK, and β-actin expressions in each group. Expression levels were quantitated by measuring band intensities using Image Lab software. The graphs indicate densitometric analyses using the expression ratios of (**B**) P-AMPK/AMPK, (**C**) P-mTOR/mTOR, and (**D**) P-ERK/ERK. The results are expressed as the mean ± SD (n = 3) in each independent experiment analyzed by one-way analysis of variance. NS: not significant; * *p* < 0.05; ** *p* < 0.01;.

The autophagy-generated mechanism in the model of CORT excess in HT22 cells was measured on the relevant key proteins by western blotting (Figure 11A). The expressions of, AMPK, P-AMPK, ERK1/2, P-ERK1/2, mTOR, P-mTOR, and β-actin were measured in the total cell lysate after CORT treatment for 3 h with or without RU486 treatment. The results showed that P-mTOR and P-AMPK were upregulated, and P-ERK was significantly downregulated in the CORT and CORT + RU486 treatment groups (Figure 11B–D).

## 4. Discussion

The study was focused on the potential mechanism of autophagy and cold exposure in the hippocampal CA1 and CA3 regions of mice, and has shown the relationships of CORT exposure, mitochondrial damage, and autophagy. In our previous results, we found that cold stress was induced following cold exposure, and that the HPA axis, hyperaction-mediated, and CORT excess secretion destroyed the homeostasis of the hippocampus and caused oxidation stress, inflammation, and neuronal apoptosis. Based on these previous results, we hypothesized that cold stress impairs hippocampus function by continually increasing the level of CORT-induced mitochondrial damage, resulting in autophagy. To confirm the hypothesis of autophagy in hippocampus following cold exposure, in vitro, we investigated the potential mechanism between CORT exposure, mitochondrial damage, and autophagy using CORT treatment of HT22 cells. The results showed that HT22 cell autophagy was induced by mitochondrial damage after CORT treatment, and that the mitochondrial damage and autophagy were greater after GR inhibition by RU486.

In vivo, the first chronic cold stress model was developed based on our previous report [8]. As previously mentioned, the CORT level was significantly increased after cold exposure, and oxidation stress occurred in the hippocampus. It is well-accepted that autophagy is the main pathway to remove damaged cell organelles to protect or reconstruct the hippocampus. There have been numerous reports that autophagy occurs in the hippocampus of mice after stress [15]. In the present report, the results showed that a marker of autophagy, LC3 II, was increased, and the relevant functional protein, p62, was downregulated following cold exposure. The completed autophagosome closure needs LC3 I transformation to LC3 II, because LC3 II is on the vesicle’s inner side and LC3 I was on the outer side. With completion of autophagosomal fusion, the LC3 II is degraded along with the cargo by ubiquitination, and the LC3 I is cleaved and recycled [16,17]. In our results, the phenomenon of autophagy may occur in order to eradicate damaged organelles. To confirm the hypothesis and relevant mechanism, we used 400 μM CORT to treat HT22 cells to develop a CORT exposure model corresponding to the time of cold exposure in vivo, showing that the GR was inhibited by RU486, which confirmed the association between CORT and autophagy. After treatment with CORT, western blots showed that the activation of GR as well as its nuclear translocation were inhibited by RU486.

The apoptosis level after CORT treatment was then measured by FITC-labeled PI and annexin V staining, indicating that the apoptosis level was increased in the CORT-treated group, and that the apoptosis level was even greater in the CORT + RU486 group. These results showed that the effect on HT22 cells from excess CORT was associated with the activation of the GR. Next, the function of mitochondrion was measured. The ATP/ADP/AMP levels were measured in the liquid phase, and the results indicated that the AMP level decreased, and that the ratio of AMP and ATP was also significantly downregulated in a time-dependent manner after CORT treatment. Previous studies have shown that the mitochondria are the energy factories of the cell, and that the AMP/ATP ratio reflects energy production [18]. A decrease in this ratio may indicate that the mitochondria were affected by CORT treatment, with this decreased trend of AMP/ATP found early in the CORT + RU486 group. The results of ∆Ψm also showed that after CORT treatment, membrane permeability was altered. In the ∆Ψm results, red emission signifies healthy mitochondria, because the healthy mitochondria are polarized, and the JC-1 taken up by such mitochondria forms aggregates. Once the ∆Ψm declines and the membrane permeability changes, the JC-1 will not accumulate in the depolarized mitochondria and is leaked into the cytoplasm, so the monomers will be green [13]. Based on this staining specificity, the JC-1 staining showed a significant decline in the ∆Ψm of HT22 cells after CORT treatment, indicating that CORT exposure resulted in significant damage to mitochondria, with the membrane permeability changes in RU486 + CORT group being even more severe. The western blotting results also demonstrated the same phenomenon. The ratio of Bcl-2/Bax significantly declined and the expression of Cyt-c increased. As previously reported, Bax forms a heterodimer with Bcl-2, then interacts with the mitochondrial voltage-dependent anion channel with increased openings leading to a loss in membrane potential and the release of cytochrome c, signifying mitochondrial damage [19,20]. Here, we confirmed that excess CORT induced mitochondrial damage, and that this damage was more severe when the GR was inhibited. The GR is located in the cytosol under physiological conditions, but it is translocated to the nucleus when it combines with CORT [21,22]. The respiration homeostasis of mitochondrial is the most important process in cell survival, and excess CORT may influence the energy cycle and cell respiration. When the GR is inhibited, the receptor is not functional, and excess CORT accumulating in the cytoplasm influences the activation of mitochondria to cause mitochondrial damage. As previously mentioned, autophagy is used to eradicate damaged cell organelles, and western blotting and MDC staining both indicated that the level of autophagy was increased after CORT treatment, which was greater when GR was inhibited. The results of electron microscopy confirmed our hypothesis that the integrity of mitochondria was compromised, and that the damage was more extensive in the CORT and RU486 + CORT group.

Finally, to confirm the hypothesis and potential mechanism of autophagy, the activations of relevant signaling pathways were measured. Western blotting showed that the phosphorylation of AMPK (Thr172) and mTOR (Ser2488) were both upregulated, while the phosphorylation of ERK was downregulated. The activation of AMPK relies on the level of AMP, so the binding of AMP and ADP, and the phosphorylation of AMPK could be induced when the ratio of AMP/ATP was unbalanced [23]. When AMPK was phosphorylated and activated, the downstream phosphorylation of mTOR was increased and the autophagy process was reversed and activated [24]. Furthermore, it has been reported that glucocorticoids can affect the kinase activity of MAPK, so excess CORT influenced cell survive by inhibiting phosphorylation of ERK.

**Figure 12:**
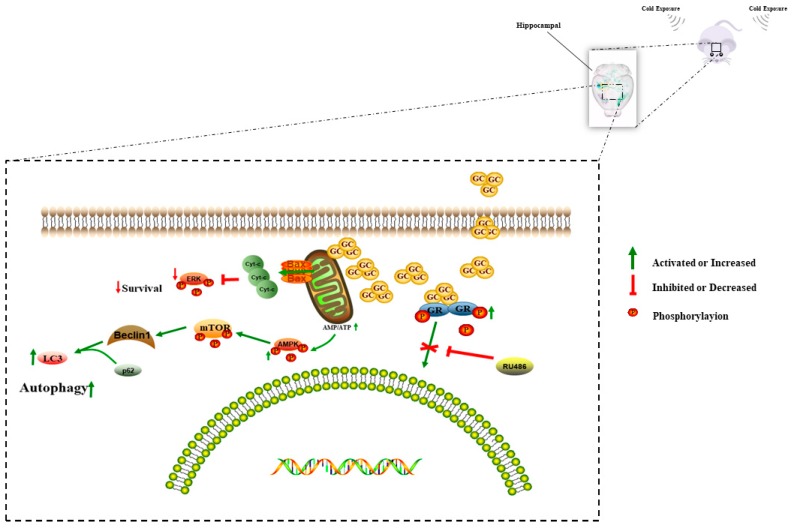
A proposed model for molecular mechanisms involving neuronal autophagy related to cold stress via 400 μM cortisol in the hippocampi of adolescent mice.

In our results, we demonstrated the difference response in hippocampus between male and female mice following cold exposure. Some research has reported there were difference reaction between distinctive gender animals under stress for the reason that the estrogen plays an important role in the process [25]. In the future, we will still focus on the potential mechanism on the impact between different gender mice following cold stress. Overall, our results indicated that repeated CORT exposure induced mitochondrial damage, which increased neuronal autophagy in the hippocampus during cold exposure (Figure 12), resulting in an increased risk of neurodegenerative disorders.

## 5. Conclusions

In our previous study, we showed that cold exposure induced the HPA axis to produce excessive CORT, which was involved in hippocampus-mediated oxidative stress. In the present study, the results were used to characterize autophagy in the hippocampus following cold exposure. Persistent neuronal stimulation of the hippocampus after CORT treatment induced mitochondrial damage and autophagy by activating the AMPK/mTOR signaling pathway, which did not rely on GRs. There was also a sex-related difference in the response to cold stress; the phenomenon of autophagy was more severe in males. These findings provided a new understanding of the underlying mechanisms of the cold stress response, which may influence the selection of animal models in future stress-related studies.
